# Optimization of Capture ELISAs for Chicken Cytokines Using Commercially Available Antibodies

**DOI:** 10.3390/ani12213040

**Published:** 2022-11-04

**Authors:** Paulina Krzysica, Loes Verhoog, Sonja de Vries, Coen Smits, Huub F. J. Savelkoul, Edwin Tijhaar

**Affiliations:** 1 Cell Biology and Immunology Group, Wageningen University & Research, De Elst 1, 6708 WD Wageningen, The Netherlands; 2 Animal Nutrition Group, Wageningen University & Research, De Elst 1, 6708 WD Wageningen, The Netherlands; 3 Trouw Nutrition, Stationsstraat 77, 3811 MH Amersfoort, The Netherlands

**Keywords:** ELISA, poultry, cytokine, interleukin, interferon

## Abstract

**Simple Summary:**

Cytokines are small proteins that are involved in the communication between immune cells. Different cytokines have distinct functions and play thus an important role in orchestrating the response of the immune system. The detection of cytokines is generally based on either mRNA analysis or the detection of protein levels via an immunoassay. However, in poultry research, tools for cytokine detection at the level of proteins are not widely available. Therefore, our aim was to develop immune assays (capture ELISAs) to measure chicken cytokine proteins using commercially available antibodies and recombinant cytokine reagents. With our optimized protocols, we were able to detect individual cytokines in culture supernatants and serum or plasma in very low, physiologically relevant levels, in the developed capture ELISAs. These capture ELISAs can easily and inexpensively be utilized and may therefore have wide applicability in immunological research for poultry.

**Abstract:**

Cytokines like interferon (IFN)-γ, interleukin (IL)-2, IL-6, IL-10, and IL-12p40 are important biomarkers for characterizing the nature and strength of immune responses. It is important to be able to quantify the cytokines at the protein level in biological samples. Quantification of chicken cytokines is generally performed on the level of messenger RNA (mRNA) by quantitative polymerase chain reaction (qPCR) because very few capture ELISAs for the quantification of chicken cytokine proteins are commercially available. Here, we describe the optimization and validation of capture ELISAs for chicken IL-2, IL-6, IL-10, IL-12p40, and IFN-γ using commercially available antibodies and reagents. First, we determined the optimal concentrations of the antibodies. We then verified the ELISAs’ performance and established that the lower limit of detection (LLOD) for all cytokines was below 32 pg/mL. The ELISAs show the same binding characteristics for recombinant and native cytokines (parallelism was <15.2% CV). Values for inter-assay variation were consistently low and mostly <20% CV. Overall, the optimized capture ELISAs are sensitive (<32 pg/mL) and reliable tools to quantify chicken cytokines. These ELISAs can easily and inexpensively be utilized in any immunological lab and may therefore have wide applicability in immunological research for poultry.

## 1. Introduction

Cytokines are small proteins that play a crucial role in the regulation of the immune system. Their molecular size usually is smaller than 30 kDa. Cytokines are produced and recognized mainly by immune cells, but can also be secreted by other cell types (e.g., epithelial cells). Cytokines serve as mediators in immune responses to infections, regulate the type of response, and the inflammatory status by attracting immune cells to the site of infection [1,2].

Different cytokines have distinct functions in orchestrating an immune response. Interferon (IFN)-γ, interleukin (IL)-2, and IL-12 are important in the induction of T helper (Th) type 1 (Th1) responses [3], which are mainly elicited against intracellular pathogens like viruses, e.g., Newcastle disease virus [4]. IL-6 has a wide range of actions and is produced at the site of inflammation and stimulates effects on T and B cells [5]. Interleukin-10, on the other hand, is an anti-inflammatory cytokine that can suppress Th1 response and induce the development of regulatory T cells (Treg) [6]. Some of these cytokines are also involved in inducing and characterizing macrophage polarization. Stimulation of macrophages by IFN-γ or lipopolysaccharide (LPS) will lead to polarization into pro-inflammatory M1 macrophages [7], which produce high levels of IL-6 [7,8] and IL-12 [7]. Stimulation of macrophages with IL-4 will result in anti-inflammatory M2 macrophages that are characterized by high production of IL-10 [7]. In chickens, IL-2, IL-6, IL-10, IL -12p40 and IFN-γ play similar roles as in mammals [4,8,9]. These five cytokines are thus implicated to play an important role in the immune responses against pathogens, defining the Th1/Th2 cytokine responses, and the nature of macrophage activation. These features make cytokines valuable biomarkers to assess the type of immune response induced by, for example, infectious diseases [10] or act as indicators for cellular activation in in vitro assays [11]. Therefore, the presence of cytokines in serum, plasma, or supernatant from cultured cells serves as an important biomarker for assessing immune function.

In avian immunology, the most commonly used tool for cytokine detection and quantification is quantitative polymerase chain reaction (qPCR) [12,13]. In this method, cytokine mRNAs are measured on a molecular level when the required product (mRNA) is artificially amplified after conversion to copy DNA (cDNA) [14]. Although the method has a number of advantages, like its quantitative feature and no need for specific antibodies [14], it does not provide information about actual concentrations of cytokines that were produced and released to the extracellular environment. It has been shown that for some cytokines, mRNA levels are not directly linked to the levels of active cytokines. Tumor necrosis factor alpha (TNF-α) for example, is first produced in an inactive form as a transmembrane protein, and only when cleaved it is released to the extracellular environment in its biologically active form [15,16]. Hence, measurements at the protein level are expected to confirm the actual production and release of cytokines. One of the methods that can measure the concentration of released cytokines at the protein level is capture ELISA. Since most cytokines are generally present at low levels (picogram/mL) in serum or plasma in vivo and have their active range in the low pg/mL to ng/mL range, these assays should be sensitive enough to quantify these low levels [17].

**Figure 1:**
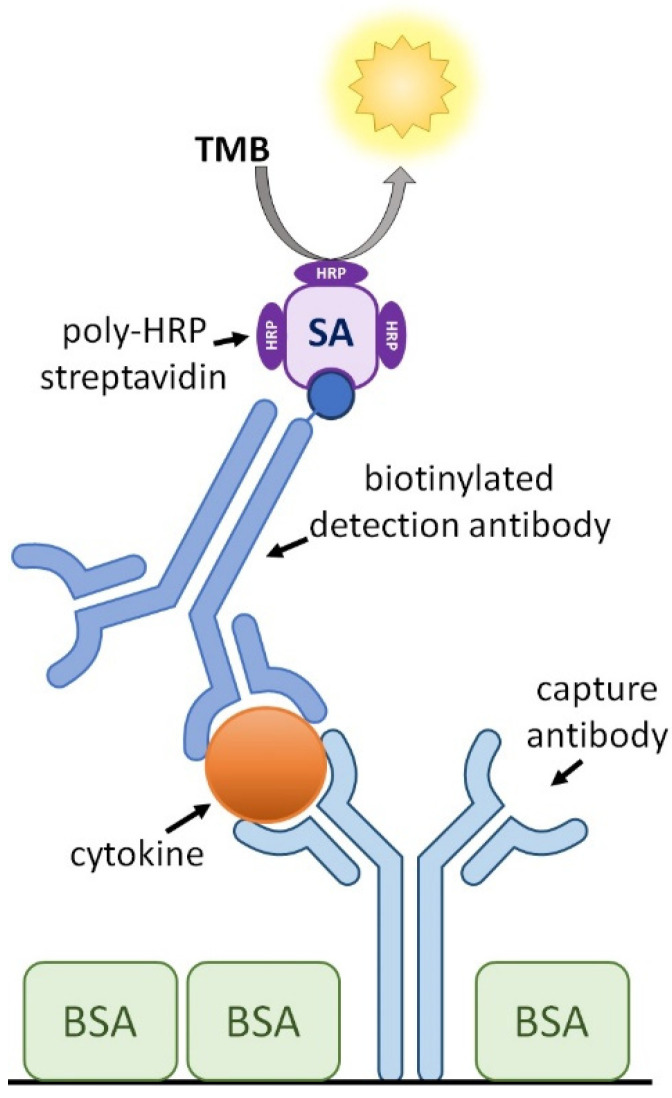
Schematic presentation of the cytokine capture ELISA. Both the capture and the detection antibodies are specific for an individual chicken cytokine protein. BSA—bovine serum albumin, SA—streptavidin, HRP—horse radish peroxidase, TMB—3,3’,5,5’-Tetramethylbenzidine.

Immunoassay in the format of a capture ELISA (Figure 1) is a sensitive assay, which uses antibody pairs specific to the proteins of interest, including cytokines, and can detect them in various sample matrices, like plasma or cell culture supernatant. For a long time, no capture ELISAs for the quantification of chicken cytokines were commercially available, except for chicken IFN-γ. This hampered research into the chicken’s immune system.

Fortunately, initiatives to optimize ELISAs and other cytokine detection assays have been undertaken in a variety of animal species [18,19], including chicken. The development of chicken-specific reagents for cytokine research has delivered a number of commercially available recombinant chicken cytokines and cytokine-specific antibodies [20]. Successful development of multiplex fluorescent-bead-based detection assay for chicken cytokines enabled simultaneous detection of IL-2, IL-10, IL-12p40, and IFN-γ [17]. However, in contrast to capture ELISA, this assay requires very specialized measuring equipment which is not widely available at each immunological laboratory. Here, based on commercially available reagents, we describe the optimization and validation of capture ELISAs for chicken IL-2, IL-6, IL-10, IL-12p40, and IFN-γ. We also provide guidelines on how to optimize the performance of capture ELISA for chicken cytokines in general when new antibodies become available. These chicken cytokine ELISAs may contribute to the toolbox for avian immunology research.

## 2. Materials and Methods

### 2.1. Ethical Approval

Samples of plasma and blood for peripheral blood mononuclear cells (PBMCs) stimulation were collected from animal experiments that were approved by the Animal Welfare Committee of Wageningen University & Research in accordance with Dutch laws and regulations on the execution of animal experiments no: AVD1040020185427 and no: AVD1040020173026.

### 2.2. Standard Capture ELISA Procedure

For all capture ELISAs (IL-2, IL-6, IL-10, IL-12p40, and IFN-γ) commercial catalog numbers of antibodies and recombinant cytokines are shown in Table 1.

ELISA high binding, 96-well, flat-bottom plates (#655061, Greiner Bio-One, Kremsmünster, Austria) were coated with 50 µL/well capture antibody (cAb) in phosphate-buffered saline (PBS; 14.4 g/L Na_2_HPO_4_ 2H_2_O, 2.4 g/L KH_2_PO_4_, 9 g/L NaCl) (at the concentrations indicated in Table 2). After gently tapping the plates to ensure equal distribution of the fluid in the wells, the plates were covered with lids to prevent evaporation and then incubated overnight at 4 °C. Subsequently, the plates were emptied and tapped dry. Wells were blocked with 100 µL 3% (*w*/*v*) Bovine Serum Albumin (BSA) Fraction V (BSAV-RO, Roche, Basel, Switzerland) in PBS. After 1 h incubation at room temperature (RT), plates were washed 3× with washing solution (0.05% (*v*/*v*) Tween20 in PBS) (#9005-64-5, Sigma-Aldrich, Saint Louis, MO, USA) and tapped dry. Recombinant cytokine (for calibration curve with two-fold dilution steps) and test samples, both diluted in dilution buffer (1.5% (*w*/*v*) BSA in PBS), were added to the plates in a volume of 50 µL/well and incubated for 1 h at RT on a microplate shaker (#444-0270, VWR, Oud-Heverlee, Belgium) at 200 RPM. In case of analyzing plasma/serum samples or samples from cell culture supernatants, heat-inactivated normal rabbit serum (#16120-107, Gibco, Invitrogen, Paisley, UK) was added to the diluted calibrators and samples to a final concentration of 0.5% (*v*/*v*). Adding rabbit serum should be checked with every new batch of antibodies because it can cause unspecific binding with new antibodies. After incubation, the plates were washed 3× with the washing solution and tapped dry. Fifty microliters of biotinylated detection antibody (dAb) in dilution buffer (see Table 2 for concentrations) were added to the wells and incubated for 1 h at RT on a microplate shaker at 200 RPM. The plates were washed 5× with the washing solution and tapped dry. For signal amplification, streptavidin conjugated to (on average) 80 horseradish peroxidase (HRP) molecules (Streptavidin poly-HRP80, #SP80C, SDT GmbH, Kraichtal, Germany) was diluted 1:5000 in PBS with the addition of 0.25% (*v*/*v*) Tween20 (#DY004, R&D Systems, Minneapolis, MN, USA) and was added to the wells in a volume of 50 µL followed by incubation in the dark for 30 min at RT on a microplate shaker at 200 RPM. ELISA plates were washed with the washing solution and tapped dry. Enhanced K-Blue TMB Substrate (#308177, Neogen, Lexington, KY, USA) (50 µL/well) was added to the plates and incubated for the appropriate time (see Table 2). During each of the previous incubation steps, the plates were covered with lids to prevent evaporation. To stop the color reaction, 50 µL/well of stop solution (0.65 M HCl in demi water) was added. The optical density at 450 nm (OD_450_), from which the OD_620_ was subtracted to correct for irregularities in the plastic of the ELISA plate, was determined in a FilterMax F5 (Molecular Devices, San Jose, CA, USA). Results were calculated using a five-parameter logistic curve fit in the program SoftMax Pro v.6.2.2 and v.7.1.0 (Molecular Devices, San Jose, CA, USA).

IFN-γ antibodies from Kingfisher Biotech Inc, Saint Paul, MN, USA (cAb PB0442C-100, dAb PBB0448C-050) did not work well during the optimizations, therefore the work was continued with Invitrogen anti-IFN-γ antibodies (Table 1).

### 2.3. Checkerboard Titrations

**Figure 2:**
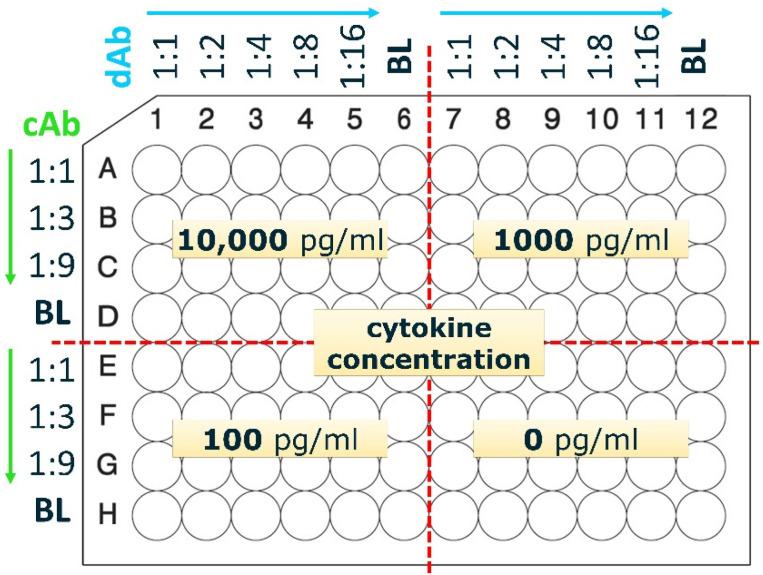
Checkerboard titration plate layout. The capture antibody (cAb) is titrated along rows and the detection antibody (dAb) along columns, as indicated. Rows D and H and columns 6 and 12 are blank (BL) and do not contain antibodies. To each of the 4 quarters of the plate one concentration of cytokine is added (10,000, 1000, 100, or 0 pg/mL). This setup allows to test the combination of multiple cAb and dAb concentrations with cytokines in one go, using one ELISA plate. Starting values (1:1) for antibodies for IL-2, IL-6 and IL-10 was 2.5 µg/mL cAb and 0.2 µg/mL dAb, for IL-12p40 was 2 µg/mL cAb and 0.02 µg/mL dAb, and for IFN-γ was 4 µg/mL cAb and 0.5 µg/mL dAb.

Checkerboard titrations (Figure 2) were performed in singlicate to assess the optimal combinations of cAb and dAb concentrations as a first step in the optimization of the ELISAs. The general protocol was as described above, with a few modifications. First, cAb dilutions were made in non-protein binding NUNC plates (#10671722, Thermo Scientific ™, Waltham, MA, USA) (Figure 2) and then 50 µL was transferred to high binding ELISA plates (Figure 2). After this coating step, the plates were blocked by a blocking buffer (3% (*w*/*v*) BSA in PBS). The plates were washed and recombinant cytokine was added to the wells (50 µL) at 4 concentrations: high (10,000 pg/mL), medium (1000 pg/mL), low (100 pg/mL) and blank (0.0 pg/mL). After incubation with the cytokines, followed by washing of the plate, the plate was incubated with dAb. Dilutions of dAb were prepared again in NUNC plates and 50 µL was transferred to the wells of ELISA plates according to the schedule shown in Figure 2. Subsequent steps were the same as described in the Standard Capture ELISA Procedure. Color development of the TMB was stopped between 5 to 30 min and stop solution was added when either the wells with the highest cytokine concentration were reaching a dark blue color or the blank wells were starting to show slight color development.

### 2.4. Validation of the Capture ELISAs

All validations shown below were performed using the optimized capture ELISA procedure (Table 2).

#### 2.4.1. Lower Limit of Detection (LLOD)

To be able to check the LLOD, 24 blanks were used next to the calibration curves for all cytokines. For blanks and calibration curves, either dilution buffer only or dilution buffer containing 5% chicken serum (in culture media) was used. The LLOD was assessed by calculating the value that corresponds to the mean OD_450-620_ results from all the blanks + 2× SD. The corresponding LLOD was interpolated using asymmetric five parametric logistic regression (Graph Pad Software Inc., San Diego, CA, USA; using standard procedures for fitting asymmetric five parametric nonlinear regression with 95% confidence interval) with Log2-transformed concentrations of the calibration curve. After interpolation, obtained Log2 value for LLOD was backtransformed.

#### 2.4.2. Matrix Effect

For testing the influence of the sample matrix on cytokine detection, recombinant cytokines were diluted in dilution buffer containing 10% (for IL-10, IL-12p40) or 20% (for IL-2, IL-6) chicken plasma (obtained from the wing vein of a healthy chicken [21]) or 5% chicken serum (#16110082, Gibco, Invitrogen, Paisley, UK) in culture media). If no standard deviation (SD) is shown, then the figure presents data from a representative experiment.

#### 2.4.3. Cell-Culture Supernatant for Validation of Parallelism

Blood from four 16-week-old WA elite pure line chickens (Hendriks Genetics, Boxmeer, The Netherlands) [22] was collected from wing veins by heparin tubes. Next, the blood was diluted with sterile PBS (#10010-015, Gibco, Invitrogen, Paisley, UK), placed on Histopaque-1.1191 (#1119, Sigma-Aldrich, Saint Louis, MO, USA) and centrifuged at 700× *g* for 40 min at RT. The interphase was collected and washed 3× with PBS at 250 g for 8 min at RT. The isolated PBMCs were resuspended in a complete culture medium (RPMI 1640, (#52400-025, Gibco, Invitrogen, Paisley, UK)) with the addition of 10% heat-inactivated chicken serum (#16110082, Gibco, Invitrogen, Paisley, UK), 1% penicillin-streptomycin (#15140122, Gibco, Invitrogen, Paisley, UK) and 1% L-glutamine (#25030024, Gibco, Invitrogen, Paisley, UK) and seeded in 96-well, flat bottom cell culture plate (#3596, Corning Inc., Corning, NY, USA) at 500,000 cells per well and stimulated with pokeweed mitogen (PWM) (#L9379, Sigma, Saint Louis, MO, USA) at 10 and 20 µg/mL for 24 h and 48 h at 41 °C, 5% CO_2_. Cell-culture supernatant was collected and stored at −80 °C for further tests.

#### 2.4.4. Parallelism

This test checks if the detection of recombinant and native cytokines is similar and requires biological samples with a high endogenous concentration of a cytokine. Therefore, samples of cell culture supernatant from PWM-stimulated PBMCs were first tested by ELISA to confirm the high concentrations of produced cytokines. Then, for each specific cytokine ELISA, samples of cell culture supernatant from four chickens were used. Each sample was diluted 2× in dilution buffer and then titrated with 2-fold dilution steps for a total of six dilutions. Each dilution was analyzed in duplicate. Measured concentrations were calculated back according to their dilution factor, and the coefficient of variation (CV) from all dilutions was calculated for each sample. Only the dilutions giving values above the limit of detection were included.

#### 2.4.5. Inter-Assay Variations

This test assesses the read-out variations between the plates. Calibration curves were calculated based on a five-parameter logistic curve and used to compare recombinant calibrator dilutions among different plates and days. Results are presented for each concentration as %CV.

## 3. Results

### 3.1. Optimization of Cytokine Capture ELISA

**Figure 3:**
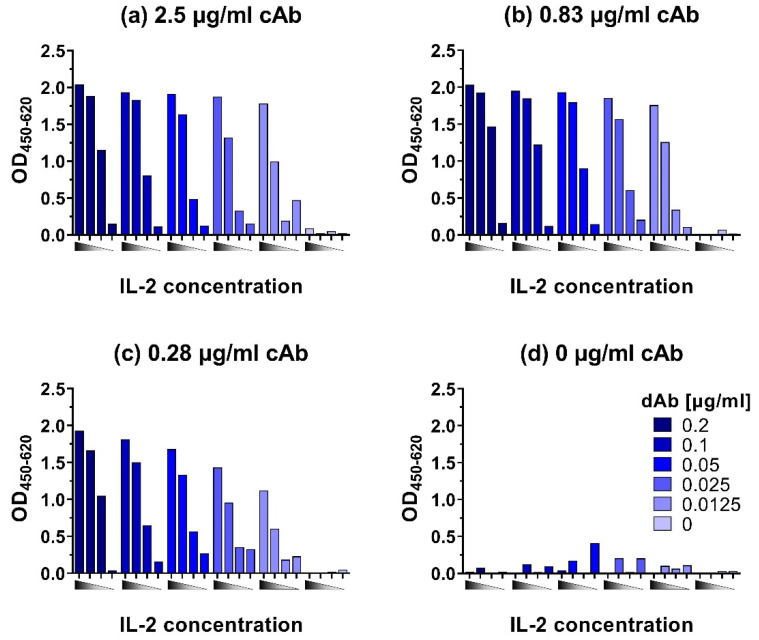
Checkerboard titration of capture antibody (cAb) at (**a**) 2.5 µg/mL, (**b**) 0.83 µg/mL, (**c**) 0.28 µg/mL, and (**d**) 0 µg/mL and titration of detection antibody (dAb) with 10,000, 1000, 100, and 0 pg/mL recombinant IL-2 from left to right represented by 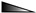. Titration was performed to check for the best combination of antibodies in a broad range of cytokine concentrations.

To develop ELISAs for the sensitive detection of chicken IL-2, IL-6, IL-10, IL-12p40, and IFN-γ, first checkerboard titrations were performed to determine suitable combinations of capture and detecting antibody concentrations for further optimization. As an example, the checkerboard titration for IL-2 is shown in Figure 3. Initial criteria for suitable combinations of cAb and dAb concentrations are an OD_450_ > 1.5 for high cytokine concentrations (up to 10,000 pg/mL), an OD_450_ < 0.1 without cytokines (negative control), and a clear distinction in signals between high (10,000 pg/mL), medium (1000 pg/mL), and low (100 pg/mL) cytokine concentrations. Most importantly, the lowest cytokine concentration tested (100 pg/mL) should have a clear signal with an OD_450_ that is approximately 2× higher than the background signal.

Checkerboard titrations were performed for all ELISAs to determine optimal concentrations of cAb and dAb, and as an example, the results of this are shown for the IL-2 ELISA (Figure 3). The highest tested cAb concentration of 2.5 µg/mL (Figure 3a) resulted in an OD_450_ signal for the lowest tested IL-2 concentration (100 pg/mL) that is at least 2× higher than the signal for the negative control (0 pg/mL). However, the difference in signals between 10,000 pg/mL and 1000 pg/mL IL-2 was only 0.3 OD_450_ when dAb concentrations of 0.2, 0.1, and 0.05 µg/mL were used, making these combinations of cAb and dAb concentrations less suitable. At 0.025 and 0.0125 µg/mL dAb the differences in signal between all three cytokine concentrations became more pronounced with a difference in absorbance of at least 0.5 OD_450_. The 0.83 µg/mL cAb concentration (Figure 3b) gave similar results as the 2.5 µg/mL cAb concentration (Figure 3a), but with a better distinction between the lowest tested IL-2 concentration of 100 pg/mL and the background (0 pg/mL). In combination with the higher dAb concentration (0.05–0.2 µg/mL) the distinction between the highest (10,000 pg/mL) and medium (1000 pg/mL) IL-2 concentrations were not very pronounced but a dAb concentration of 0.83 µg/mL in combination with 0.0125–0.025 µg/mL enabled a clear distinction between all four IL-2 concentrations. A good distinction in OD_450_ values for the different IL-2 concentrations was also obtained with a cAb concentration of 0.28 µg/mL in combination with the 0.05–0.2 µg/mL dAb (Figure 3c). In Figure 3d, where no cAb was used, in some cases, a relatively high background signal was detected. We analyzed the reason for this unspecific binding and it turned out that in some cases streptavidin poly-HRP80 showed an affinity for Tween-20 bound to the plate. The addition of Tween-20 to the streptavidin poly-HRP80 containing buffer blocked this interaction, which resulted in a reduction of the unspecific background in ELISAs (Supplementary Material Figure S1).

The initial criteria for suitable cAb and dAb combination were OD_450_ > 1.5 for cytokine concentrations up to 10,000 pg/mL, an OD_450_ < 0.1 for the negative control, a clear distinction in signals between 10,000 pg/mL, 1000 pg/mL, and 100 pg/mL cytokine and for the lowest cytokine concentration a signal that is clearly above background. Overall, combinations of cAb concentrations between 0.83 and 2.5 µg/mL with dAb concentrations between 0.0125 and 0.05 µg/mL would fulfill these criteria for suitable cAb and dAb concentrations, as would 0.28 µg/mL cAb in combination with 0.05–0.2 µg/mL dAb. All these combinations would therefore form suitable combinations for further optimization of the IL-2 capture ELISA but the use of the lower concentration of cAb (0.28 µg/mL) would result in the most economic use. Hence, the combination of 0.28 µg/mL cAb with 0.1 µg/mL dAb was tested further with a full IL-2 concentration range of 9.8–5000 pg/mL.

**Figure 4:**
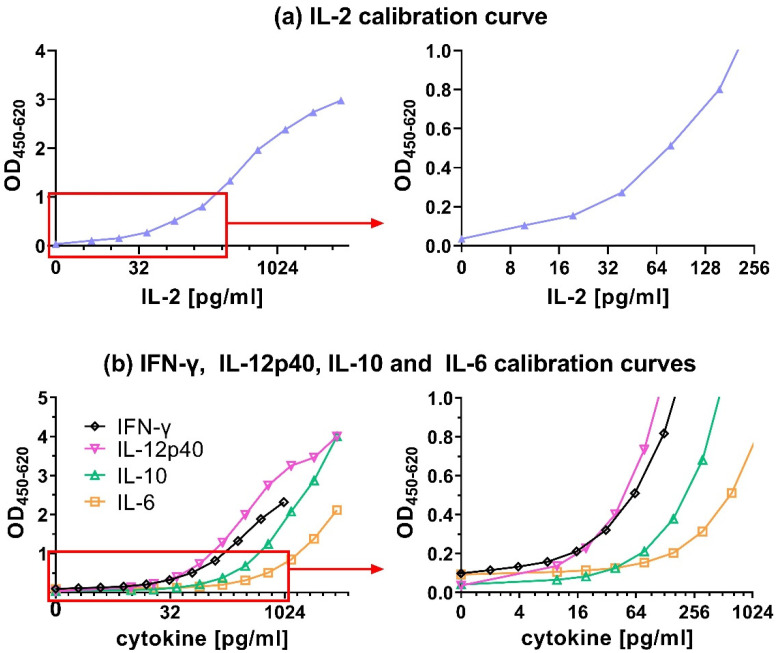
Calibration curves for recombinant chicken (**a**) IL-2, (**b**) IL-6, IL-10 and IL-12p40 ranging from 9.8 to 5000 pg/mL, and IFN-γ ranging from 2 pg/mL to 1000 pg/mL presented at the full concentration range (left graphs) and the lower concentration range up to 1.0 OD (right graphs). All results represent absorbance signals.

A combination of 0.28 µg/mL cAb with 0.1 µg/mL dAb resulted in a good IL-2 calibration curve in which all the values between 9.8 and 5000 pg/mL could be distinguished from each other (Figure 4a). Furthermore, other criteria were fulfilled like a high OD_450_ value for the maximum tested IL-2 concentration of well above 1.5 (i.e., 2.97), an OD_450_ < 0.1 for the negative control (i.e., 0.03) and the lowest tested IL-2 concentration of 9.8 pg/mL was with an OD_450_ of 0.1 clearly distinguishable from the background signal of 0.03. This same procedure used for IL-2 was also followed for all the other chicken cytokine ELISAs, to obtain optimized combination of cAb and dAb concentrations (Table 2) resulting in the calibration curves shown in Figure 4b. These selected combinations were used in all successive ELISA tests.

### 3.2. Detection Limits of Capture ELISAs

The LLOD, defined as the lowest signal that can still be distinguished from the background signal, determines the sensitivity of the ELISA. It is the cytokine concentration of the calibration curve that corresponds with the mean OD_450_ results from all blanks + 2× SD. The LLOD for all ELISAs was between 1–32 pg/mL (Table 3). For IFN-γ, the OD_450_ signal of blank samples + 2× SD was below the lowest tested concentration of the calibration curve (1.95 pg/mL). Hence, the LLOD for IFN-γ was < 1.95 pg/mL. This value was followed by IL-12p40 (3.4 pg/mL), IL-2 (12 pg/mL), IL-6 (15 pg/mL) and finally IL-10 (32 pg/mL) (Table 3).

The LLOD values can vary in samples in a more complex matrix like cell culture supernatant with 10% serum. Therefore, the LLOD was also determined in the presence of 5% chicken serum (Supplementary Material Table S1) to represent twice diluted cell culture supernatant samples that could be tested by ELISA after, e.g., cell stimulation experiments. The LLOD for IFN-γ in the presence of 5% chicken serum was determined as 1 pg/mL. An increase in LLOD levels of 3x was noted for IL-12p40 (10 pg/mL) and 1.5× for IL-6 (23 pg/mL)(Supplementary Material Table S1). For the IL-10 ELISA, the LLOD level was almost unchanged (33 pg/mL) in the presence of serum. Surprisingly, the LLOD for IL-2 was approximately 2× lower (6 pg/mL) than determined earlier without serum. Although the addition of 5% serum somewhat influenced LLOD levels, all LLODs of the different ELISAs were below 32 pg/mL.

The calibration curves were not checked for higher cytokine concentrations than those mentioned, and therefore the upper limits of detection (ULOD) were not determined. Clearly, the calibration curves did not reach saturation, indicating that the ULOD will be higher than the highest tested cytokine concentrations, except for IL-2 which reached saturation between 1250 pg/mL and 5000 pg/mL (Figure 4).

### 3.3. Matrix Effect and Cytokine Detection

**Figure 5:**
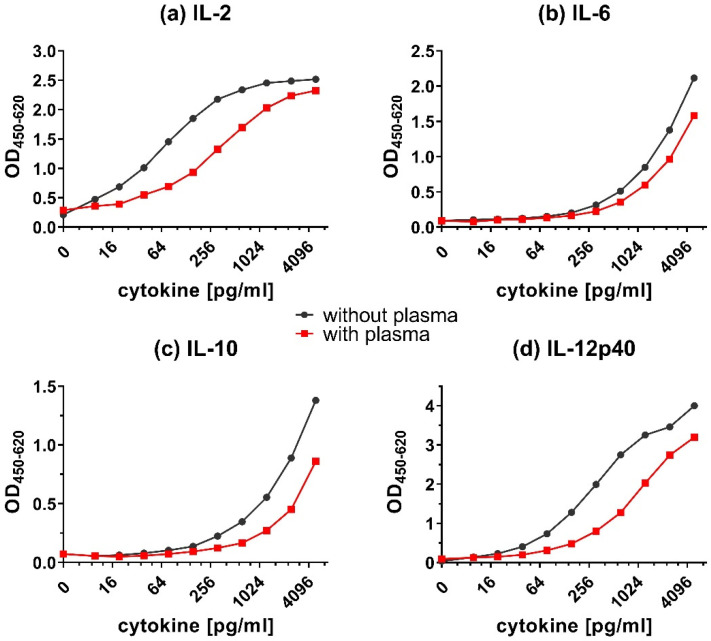
Matrix effect on the detection of cytokines in presence of plasma. Calibration curves for (**a**) IL-2, (**b**) IL-6, (**c**) IL-10, and (**d**) IL-12p40 in dilution buffer with or without plasma. The capture ELISA was performed with a range of 9.8–5000 pg/mL of recombinant cytokines in dilution buffer (1.5% BSA in PBS) only (without plasma) or containing additional chicken plasma (with plasma).

In addition to effects on the LLOD values, the matrix of the samples, e.g., the presence or absence of serum or plasma, can also influence the detection of cytokines. Components in the matrix could cross-react with the capture or detecting antibodies and mask the detection of cytokines. Adding plasma to the calibration curves (Figure 5) caused a decrease in the OD_450_ signal of all detected cytokines. This signal reduction was strongest for IL-12p40 (Figure 5d), followed by IL-2 (Figure 5a). The detection of IL-6 (Figure 5b) and IL-10 (Figure 5c) were only modestly affected by the addition of plasma.

**Figure 6:**
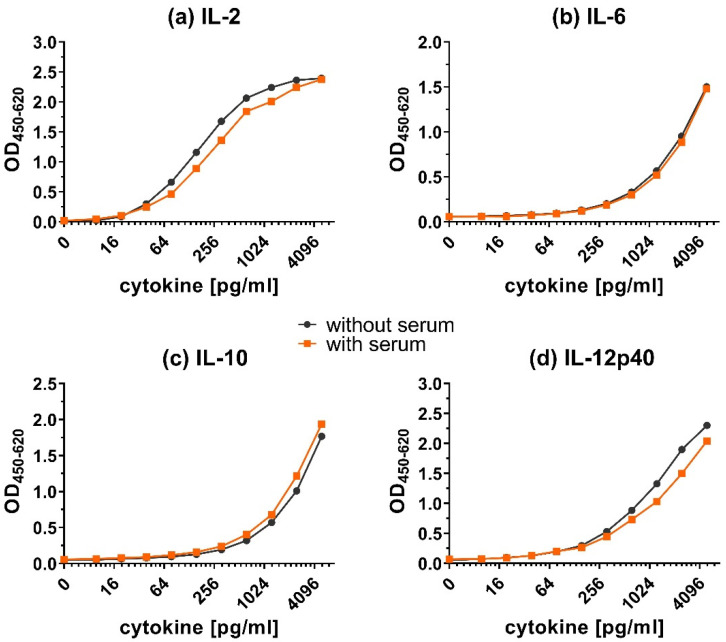
Matrix effect on the detection of cytokines in presence of serum. Calibration curves for (**a**) IL-2, (**b**) IL-6, (**c**) IL-10 and (**d**) IL-12p40 in dilution buffer with or without serum. The capture ELISA was performed with a range of 9.8–5000 pg/mL of recombinant cytokines in dilution buffer (1.5% BSA in PBS) only (without serum) or containing additional chicken plasma (with serum).

The addition of serum had little effect on the calibration curves (Figure 6). The detection of IL-12p40 and IL-2 was slightly reduced in the presence of serum, but much less than with plasma. The signal of IL-6 and IL-10 did not decrease after adding chicken serum.

Overall, adding plasma (Figure 5) resulted in a shift of calibration curves to the right for all tested cytokines. However, adding serum (Figure 6) resulted in very little to no shift of the calibration curves. Although the addition of plasma did reduce the obtained OD values, the shape of the calibration curves was preserved.

### 3.4. Binding Characteristics of Native Cytokines

**Figure 7:**
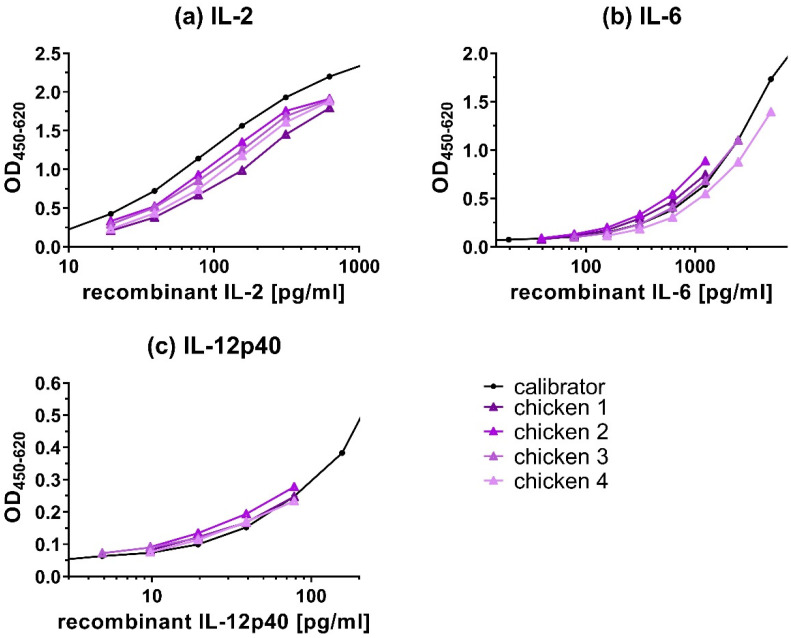
Parallelism of recombinant and native (**a**) IL-2, (**b**) IL-6, and (**c**) IL-12p40 from stimulated PBMCs. As a source of native cytokines, cell culture supernatant of pokeweed mitogen (PWM) stimulated peripheral blood mononuclear cells (PBMCs), isolated from four different chickens, was used. Of these samples, 2× dilutions (chicken 1–4) were tested by capture ELISA and compared with a 2× dilution curve of recombinant cytokines (calibrator). Triangles represent the sample dilutions (and not cytokine concentrations) in comparison to their respective recombinant cytokine (calibration curve). The sample titrations show a high degree of parallelism with the calibration curves of the recombinant cytokines, indicating the similar binding characteristics of the capture ELISAs for the recombinant and native cytokines. Results represent OD_450-620_ signal.

Experiments were performed to assess if native cytokines showed similar binding characteristics in the capture ELISAs as the recombinant cytokines used for the calibration curve. If native and recombinant cytokines show similar binding characteristics, then dilutions of both types of cytokines should result in parallel curves. Culture supernatants of PWM-stimulated chicken PBMCs were used as a source of native cytokines. To evaluate the parallelism for IL-2, IL-6, and IL-12p40 (Figure 7), samples’ readouts were plotted on the graph for visual comparison to the calibration curves and %CV was calculated from measured cytokine concentrations (Table 4). The titration curves of the samples were similar to the calibration curves of recombinant IL-2 (Figure 7a), IL-6 (Figure 7b), and IL-12p40 (Figure 7c), indicating a high degree of parallelism.

The CV of the titration of four samples (Table 4) ranged between 7.42%–15.14 % for IL-2, 6.05%–9.02% for IL-6 and 11.16%–15.18% for IL-12p40.

For the cytokines IL-2, IL-6, and IL-12p40 (Figure 7), curves run in parallel and %CV of cytokine concentrations between different sample dilutions, calculated back to the undiluted sample, were low (<16%) indicating similar binding characteristics of native and recombinant cytokines by the capture ELISAs. These results indicate that the detection of cytokines from the samples was not influenced by the sample’s dilutions. For IL-10 and IFN-γ capture ELISAs, parallelism tests could not be performed, because samples with a high enough endogenous concentration of these cytokines were not available.

### 3.5. Inter-Assay Variations

Inter-assay variations show the level of read-out variations of the same calibration curve tested on different ELISA plates and on different days.

Inter-assay variations for most of the analyzed cytokine concentrations were low for the IL-2, IL-6, and IL-10 capture ELISAs, with values generally well below 20% CV and acceptable for the IL-12p40 ELISA with <30% CV for most tested concentrations (Table 5). At the higher range of tested cytokine concentrations, the inter-assay variation becomes larger (>30% CV) for IL-2 (>625 pg/mL) and IL-12p40 (at 5000 pg/mL). The inter-assay variation also becomes larger (>30% CV) at concentrations <19.5 pg/mL (IL-6 and IL-12p40) or <39.1 pg/mL (IL-10). The capture ELISAs show therefore inter-assay variation of <30% CV for the following detection ranges: 9.8–625 pg/mL for IL-2, 19.5–5000 pg/mL for IL-6, 39.1–5000 pg/mL for IL-10 and 19.5 to 2500 pg/mL for IL-12p40. This validation was not performed for IFN-γ ELISA, because the number of tests for inter-assay variation was not sufficient to properly calculate %CV. This was due to the change of the source of cAb and dAb from Kingfisher to Invitrogen that had to be made for this cytokine during the research.

## 4. Discussion

In this paper, by using commercially available antibodies and recombinant cytokines, we showed the optimization and validation of capture ELISAs for chicken IL-2, IL-6, IL-10, IL-12p40, and IFN-γ. These tools enable low-cost and easy detection of chicken cytokines from culture supernatants and other biological samples.

Capture ELISA protocols were optimized to obtain the highest sensitivity with the lowest antibody concentrations to reduce costs. Checkerboard titrations were used as the first test to screen for the best combination of antibody concentrations, as often it can be lower than the concentrations advised by the manufacturer [23]. Such a checkerboard titration is advised to be repeated with every new batch of antibodies if it does not perform similarly to the previous batch.

These ELISAs make use of commercially available and affinity purified polyclonal capture and detection antibodies that are all generated in rabbits. To distinguish the capture from the detecting antibody, the detecting antibody is biotinylated by the manufacturer. For better sensitivity and strength of the signal, we used this biotinylated dAb in combination with streptavidin conjugated with on average 80 HRP molecules. The biotin-streptavidin bond is the strongest non-covalent bond known in nature [24] and is highly specific [25], which reduces the non-specific signal in the assay. To further increase the detection signal a highly sensitive TMB substrate was used. Due to the strong amplification of the detection signal, it is essential that the ELISA plates are very well washed, in particular after the dAb and streptavidin-polyHRP80 incubation steps, because any of these molecules that bound nonspecifically will result in an increase in background signal and a subsequent reduction in sensitivity.

Another approach to detecting released chicken cytokines, which allows for the detection of several different cytokines simultaneously, is called Bio-Plex assay and was developed recently [17]. However, this method requires special equipment and beads that either must be prepared or purchased (#GCYT1-16K, Millipore, Burlington, MA, USA). Capture ELISAs only require a spectrophotometric ELISA plate reader that will generally be available in most laboratories performing (basic) immunological research and can be performed at low costs.

The LLOD indicates the lowest cytokine level that can be distinguished from the background [26]. For all capture ELISAs that are presented in this paper, the LLOD is lower than 32 pg/mL. The sensitivity of these assays allows the measurement of low, physiologically relevant, levels of cytokines in biological samples [27]. In addition, when sensitivity was tested in the presence of chicken serum, the LLOD values were hardly affected.

To verify the influence of the matrix on cytokine detection, chicken plasma or serum was added to the calibration curves. In the presence of these two more complex matrixes, the calibration curves shifted to the right, resulting in lower OD values at the same concentration compared with calibration curves in dilution buffer only. This shift indicates a masking effect of the matrix on detected cytokines [28]. This was not observed for the curves of IL-10 and IL-6 spiked with serum. The curve of the IL-10 calibrator shifted even to the left, thereby resulting in higher OD values. This might be caused by the presence of endogenous IL-10 in serum. The detection of IL-6 was not influenced by this matrix. Nevertheless, in all cases, the shape of the curves remained the same, which means that the signal was still specific for the detected cytokines and that the cytokine’s relative concentration can still be measured. For accurate quantification of absolute concentrations of cytokines in these more complex matrixes, the calibration curve has to be prepared in the same matrix [29].

The IL-2, IL-6, and IL-12p40 capture ELISAs showed very good parallelism of samples containing native cytokines produced by PWM-stimulated PBMCs with CV values that did not pass 15.2%. Although acceptance criteria for parallelism’s values can vary, our ELISAs fall in the range of the most strict criteria—below 20% CV [26,30]. This indicates that titration of the samples does not affect their detection and binding capacities to the ELISA antibodies [26].

For inter-assay variations, the majority of the tested cytokine concentrations for IL-2, IL-6, and IL-10 between 5000 pg/mL and the LLOD concentration were below 20% CV, demonstrating low variation between plates and good assay-to-assay repeatability [31]. Although values for IL-12p40 were somewhat higher than in other ELISAs, the majority of CV values were still below 30%. Out of all the ELISAs, only a few concentrations were above 30% CV, and among these were the cytokine concentrations below the LLOD.

## 5. Conclusions

The optimized capture ELISAs for chicken cytokines are sensitive assays that can reliably detect native cytokines with a low plate-to-plate variation. These assays can be used to compare cytokine concentrations between biological samples, however, to determine absolute concentrations, a suitable matrix should be added to the calibration curve. These ELISAs can easily and inexpensively be produced in any immunological lab and may therefore have wide applicability in immunological research in poultry.

## Figures and Tables

**Table 1:** Reagents used for the chicken cytokine capture ELISAs and their catalog numbers.

	IL-2	IL-6	IL-10	IL-12p40	IFN-γ
Capture antibody (cAb)	PB0387C-100, KB	KP1113C-100, KB	KP1116C-100, KB	PB0435C-100, KB	CAC1233, IVG
Recombinant cytokine	RP0063C-005, KB	RP0299C-005, KB	RP0018C-005, KB	RP0289C-005, KB	RP0929C-100, KB
Biotinylated detection antibody (dAb)	PBB0395C-050, KB	KPB1114C-050, KB	KPB1117C-050, KB	PBB0436C-050, KB	CAC1233, IVG

All capture and biotinylated detection antibodies from Kingfisher Biotech are rabbit polyclonal antibodies raised and affinity purified against the recombinant chicken cytokines that were produced in yeast (Pichia). The matched antibody pair from Invitrogen consists of mouse monoclonal antibodies. KB: Kingfishcer Biotech, Inc. Saint Paul, MN, IVG: Invitrogen, Inchinnan, UK.

**Table 2:** Working concentrations of capture antibody (cAb) and detection antibody (dAb) and incubation time for TMB substrate.

	IL-2	IL-6	IL-10	IL-12p40	IFN-γ
Concentration of cAb [µg/mL]	0.28	2.5	2	0.67	2
Concentration of dAb [µg/mL]	0.1	0.2	0.5	0.2	0.25
TMB incubation time [min]	5	19	30	15	10

**Table 3:** Lower limit of detection (LLOD).

	IL-2	IL-6	IL-10	IL-12p40	IFN-γ
LLOD [pg/mL]	12.01	15.11	31.82	3.39	<1.95

The LLOD concentrations were calculated by interpolating from calibration curve the values of the mean OD_450-620_ + 2 × SD of 24 blank samples containing dilution buffer only.

**Table 4:** Parallelism of samples containing native cytokines.

	Parallelism between Recombinant and Native Cytokines [%CV]		
	IL-2	IL-6	IL-12p40
Sample 1	7.42	9.02	12.74
Sample 2	15.14	6.37	11.16
Sample 3	10.78	7.51	15.18
Sample 4	7.65	6.05	14.89

Parallelism of the samples was assessed by titrating supernatant of pokeweed mitogen (PWM) stimulated peripheral blood mononuclear cells (PBMCs). Coefficient of variation (%CV) was calculated from samples’ titration that was calculated back according to their dilution factors. All dilutions were run in ELISA in duplicates.

**Table 5:** Inter-assay (between plates) variations for calibration curve concentrations.

	Inter-Assay Variation (%CV)			
Cytokine Concentration [pg/mL]	IL-2	IL-6	IL-10	IL-12p40
5000	32.8	5.1	0.5	37.5
2500	23.2	13.9	1.5	13.7
1250	55.9	5.8	2.2	28.0
625	17.8	3.0	4.3	27.8
312.5	10.8	4.9	5.8	27.7
156.3	9.2	8.0	5.0	27.9
78.1	5.5	10.0	15.4	28.0
39.1	5.4	13.4	23.7	27.9
19.5	7.8	19.7	202.8 *	29.1
9.8	16.2 *	54.8 *	95.7 *	45.7

All values come from OD_450-620_ signal that was calculated back and then compared with values from calibration curves from other plates and days. *—values below LLOD levels

## Data Availability

The data presented in this study are openly available in 4TU.ResearchData at https://doi.org/10.4121/21494520.

## Supplementary Materials

The following supporting information can be downloaded at: http://www.mdpi.com/2076-2615/12/21/3040/s1, Figure S1: Checkerboard titration of capture antibody (cAb) at (a) 2.5 µg/ml, (b) 0.83 µg/ml, (c) 0.28 µg/ml and (d) 0 µg/ml and titration of detection antibody (dAb) with 1000, 100, 10 and 0 pg/ml recombinant IL-2 from left to right represented by ; Table S1: Lower limit of detection (LLOD) in 5% chicken serum which is simulating more complex matrix of cell culture supernatant.
