# Supplementary material for: Optimization of Capture ELISAs for Chicken Cytokines Using Commercially Available Antibodies
Source: Animals (Basel). 2022 Nov 4;12(21):3040. doi: 10.3390/ani12213040 (PMC9658146; doi:10.3390/ani12213040)
Supplement: Supplementary file 1 [file animals-12-03040-s001.zip › animals-1966508-supplementary.pdf]

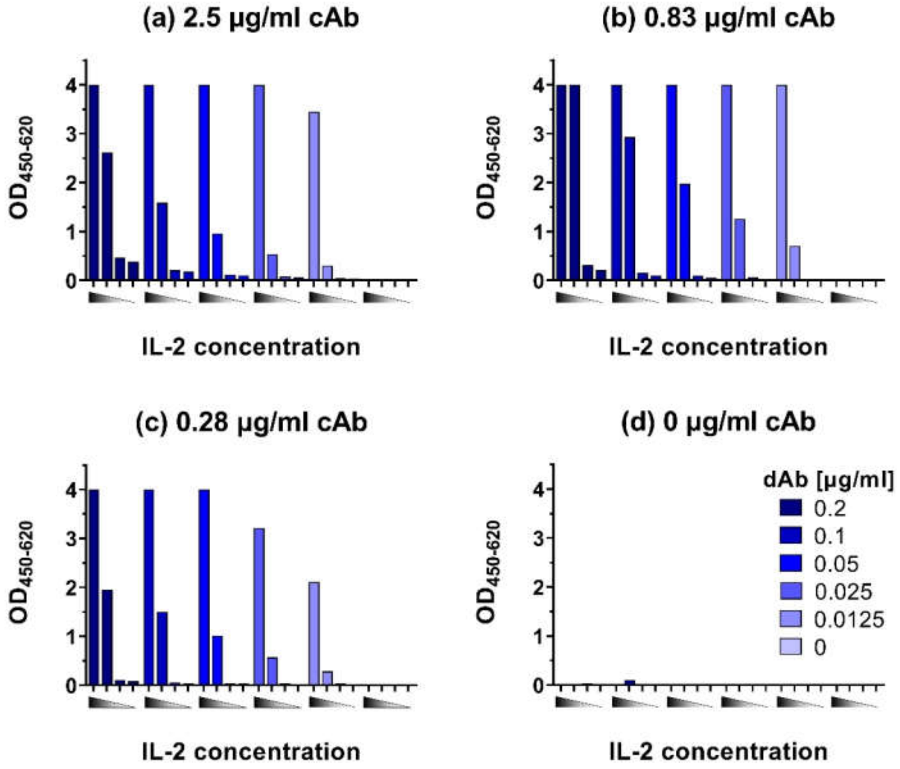

Figure S1. Checkerboard titration of capture antibody (cAb) at (a) 2.5 µg/ml, (b) 0.83 µg/ml, (c) 0.28 µg/ml and (d) 0 µg/ml and titration of detection antibody (dAb) with 1000, 100, 10 and 0 pg/ml recombinant IL-2 from left to right represented by ◣. Titration was performed to check for the best combination of antibodies in broad range of cytokine concentrations in presence of Tween20 in streptavidin-polyHRP80.

Table S1. Lower limit of detection (LLOD) in 5% chicken serum which is simulating more complex matrix of cell culture supernatant

	**IL-2**	**IL-6**	**IL-10**	**IL-12p40**	**IFN-γ**
LLOD [pg/ml]	5.65	22.87	32.83	10.41	1.03

The LLOD concentrations were calculated by interpolating from calibration curve the values of the mean $OD_{450\text{-}620}$ + 2×SD of 24 blank samples containing dilution buffer only.
